# Longer and Less Overlapping Food Webs in Anthropogenically Disturbed Marine Ecosystems: Confirmations from the Past

**DOI:** 10.1371/journal.pone.0103132

**Published:** 2014-07-30

**Authors:** Fabiana Saporiti, Stuart Bearhop, Laura Silva, Damián G. Vales, Lisette Zenteno, Enrique A. Crespo, Alex Aguilar, Luis Cardona

**Affiliations:** 1 Department of Animal Biology and Institut de Recerca de la Biodiversitat (IRBio), Faculty of Biology, University of Barcelona, Barcelona, Spain; 2 Centre for Ecology and Conservation, School of Biosciences, University of Exeter, Cornwall Campus, Penryn, United Kingdom; 3 Laboratory of Marine Mammals, Centro Nacional Patagónico - Consejo Nacional de Investigaciones Científicas y Técnicas (CENPAT-CONICET), Puerto Madryn, Argentina; Institut Pluridisciplinaire Hubert Curien, France

## Abstract

The human exploitation of marine resources is characterised by the preferential removal of the largest species. Although this is expected to modify the structure of food webs, we have a relatively poor understanding of the potential consequences of such alteration. Here, we take advantage of a collection of ancient consumer tissues, using stable isotope analysis and SIBER to assess changes in the structure of coastal marine food webs in the South-western Atlantic through the second half of the Holocene as a result of the sequential exploitation of marine resources by hunter-gatherers, western sealers and modern fishermen. Samples were collected from shell middens and museums. Shells of both modern and archaeological intertidal herbivorous molluscs were used to reconstruct changes in the stable isotopic baseline, while modern and archaeological bones of the South American sea lion *Otaria flavescens*, South American fur seal *Arctocephalus australis* and Magellanic penguin *Spheniscus magellanicus* were used to analyse changes in the structure of the community of top predators. We found that ancient food webs were shorter, more redundant and more overlapping than current ones, both in northern-central Patagonia and southern Patagonia. These surprising results may be best explained by the huge impact of western sealing on pinnipeds during the fur trade period, rather than the impact of fishing on fish populations. As a consequence, the populations of pinnipeds at the end of the sealing period were likely well below the ecosystem's carrying capacity, which resulted in a release of intraspecific competition and a shift towards larger and higher trophic level prey. This in turn led to longer and less overlapping food webs.

## Introduction

Human activities have altered most of the coastal marine ecosystems of the world over many centuries, causing reductions in population sizes, shifts in geographic ranges, and losses of diversity, biomass, and ecosystem functioning [1], [2], [3], [4], [5], [6]. Both aboriginal and industrial exploitation of marine resources are characterised by the preferential removal of the largest species [3], [7], [8], [9], [10], a process thought to shorten size-structured marine food webs [4], [11], [12]. However, so far, megafaunal exploitation has generally resulted in population collapse and top predator rarity, rather than in true extinctions [13], [14], [15]. The difference between rarity and extinction is relevant, because the extinct species no longer belong to the local food web, whereas the scarce ones remain, although it has been argued that these can be considered functionally extinct [10], [16].

To understand the actual relevance of both extinction and scarcity of formerly abundant megafauna species, we need to assess their role in the pristine ecosystems in which they have evolved. This can be achieved by studying marine protected areas (e.g. [17]) but the influence of the human disturbed matrix in which they are embedded is difficult to control. Furthermore, marine reserves are often too small to support viable populations of large top predators or encompass entire foraging ranges [18] and have not been protected for long enough to guarantee full recovery [19]. A second approach is through ecosystem modelling (e.g. [20], [21]), but this can be hampered by the limited perspective of contemporary data [3], the difficulties of model parameterisation [22] and unknown changes in the diet and foraging behaviour of predators through time (e.g. [23], [24]). A third approach consists of using ancient biological material, such as bones and shells, to reconstruct trophic relationships in a time span prior to the anthropogenic alteration of marine ecosystems [3].

The mechanisms and underlying causes of a number of cases of population collapses or extinctions have been clarified by analysing the fossil and zooarchaeological records (e.g. [25]). However, this method brings little information about past diets, trophic levels and food-web structure. The development of stable isotope analyses and quantitative methods for analysing the food-web topology [26], [27], [28] allows the reconstruction of past trophic relationships based on ancient biological material [29], since the stable isotope ratios in consumer tissues represent those of their diet in a predictable manner [30], [31]. The marine ecosystems off Argentina have suffered major anthropogenic changes during the past two centuries. Firstly, European sealers locally extirpated the South American fur seal (*Arctocephalus australis*) during the 19^th^ century and were at least in part responsible for a decline in South American sea lion populations (*Otaria flavescens*) during the first half of the 20^th^ century [32], [33], [34], [35], [36]. When exploitation ceased most populations of this species had been reduced to <10% of the pre-exploitation numbers and their recovery did not begin until the early 1990s in Argentina, after several decades of stagnation [34], [37], [38]. As a result, the current populations of both species are still well below pre-exploitation numbers [34], [39], [40], [41], [42]. Furthermore, fisheries targeting large demersal fishes were established in Río de la Plata and northern Patagonia in the 1970s, causing a population decline in several large benthic predatory fish species [43], [44], [45], [46]. Finally, the Argentine population of the Magellanic penguin (*Spheniscus magellanicus*) increased during the 20^th^ century, both in number of individuals and geographic range [47], perhaps due to the decline of competitors and predators because of the exploitation of pinnipeds [48].

However, the European sealers and fishermen were not the first humans to exploit the marine resources off Argentina. Aboriginal hunter-gatherers began to exploit the local populations of fur seals, sea lions and other marine species during the middle Holocene [49], [50], [51], [52], [53], [54]. Even though the general opinion is that the impact on the populations of pinnipeds was minimal [49], [55]. More recently, Zangrando and colleagues [56] argued that human pressures on fur seals in the Beagle Channel during the late Holocene might have resulted in demographic and ecological changes, based on evidence from stable isotope analyses and the decreasing age and size of hunted individuals. However, these results remain inconclusive because potential variations of the stable isotope baseline through time were not accounted for [57]. The zooarchaeological record preserved in the hunter-gatherers middens offers an excellent opportunity to reconstruct the structure of ancient marine food webs (while accounting for potential changes in stable isotopic baselines) in the South-western Atlantic and compare it with that of modern food webs.

In this paper, we use the stable isotopes of carbon and nitrogen from the shells of mollusc shells to set the stable isotopic baseline and from bones of marine mammals and penguins to reconstruct the changes in the topology of coastal food webs from central-northern Patagonia and southern Patagonia through the second half of the Holocene. Through this, we aim to assess whether the structure of the food web has changed as a consequence of human exploitation during the studied period.

## Material and Methods

### Ethics Statement

Permits to collect modern samples (Table 1 and S1) were issued by the “Dirección de Fauna y Flora Silvestre”, and the “Dirección de Areas Protegidas”, both from the Province of Chubut. The zooarchaeological samples come from previous fieldwork carried out by Julieta Gómez Otero, Florencia Borrella, Martín Serrán and Lorena Peralta in Golfo San Matías and Península Valdés (central-northern Patagonia), Eduardo Moreno in Santa Cruz (southern Patagonia) and Ernesto Piana, Luis Orquera, Angie Tivoli and Francisco Zangrando in the Beagle Channel (Tierra del Fuego). All the samples used for this study come from stranded animals, died naturally (except for mussels and limpets), or from archaeological remains (shell middens). All specimen numbers and repository information are shown in Tables 2 and 3, and in Tables S2 and S3. Due to the low proportion of organic matter present in the archaeological shells, the entire valve was used to undertake isotopic analyses. However, samples of the same ages are available to allow the work to be reproducible.

**Table 1:** Samples used to reconstruct the modern food webs.

Species	Area	N
**Herbivores**		
*Mytilus edulis*	Río de la Plata	5
*Aulacomya atra atra*	Northern Patagonia	7
*Aulacomya atra atra*	Southern Patagonia	5
*Siphonaria lessoni*	Río de la Plata	5
*Nacella magellanica*	Northern Patagonia	5
*Nacella magellanica*	Southern Patagonia	5
**Top Predators**		
*Arctocephalus australis*	Río de la Plata	60
*Arctocephalus australis*	Northern Patagonia	29
*Arctocephalus australis*	Southern Patagonia	7
*Otaria flavescens*	Río de la Plata	19
*Otaria flavescens*	Northern Patagonia	36
*Otaria flavescens*	Southern Patagonia	41
*Spheniscus magellanicus*	Río de la Plata	20
*Spheniscus magellanicus*	Northern Patagonia	20
*Spheniscus magellanicus*	Southern Patagonia	40

**Table 2:** Archaeological samples from northern-central Patagonia.

Species	N	Laboratory ID	Archaeological site	Age (^14^C yr BP)	Reference	Repository information
**Herbivores**						
*Aulacomya atra atra*	3	A7-cC1, C4, C5	Los Abanicos 1	380±60	[105]	CENPAT, Puerto Madryn (Argentina)
*Aulacomya atra atra*	2	A8-cC2, C4	Las Ollas conchero 1	610±60, 640±60	[52]	CENPAT, Puerto Madryn (Argentina)
*Aulacomya atra atra*	5	A5-cC1, C2, C3, C4, C5.	Playas Las Lisas 2-perfil 1	2140±50	[52]	CENPAT, Puerto Madryn (Argentina)
*Aulacomya atra atra*	2	A11-cC3, C4	Cracker 8-Nivel 3	5200±70	[52]	CENPAT, Puerto Madryn (Argentina)
*Nacella magellanica*	5	A7-cL1, L2, L3 L4, L5.	Los Abanicos 1	380±60	[105]	CENPAT, Puerto Madryn (Argentina)
*Nacella magellanica*	5	A10-cL1, L2, L3, L4, L5.	Ecocentro Fogón 3	850±150	[52]	CENPAT, Puerto Madryn (Argentina)
*Nacella magellanica*	4	A4- cL1, L3, L4	Playa Las Lisas 2-conchero 2	2600±60	[52]	CENPAT, Puerto Madryn (Argentina)
*Nacella magellanica*	2	A11-cL1, L2	Cracker 8-Nivel 3	5200±70	[52]	CENPAT, Puerto Madryn (Argentina)
**Predators**						
*Arctocephalus australis*	3	F1 18, F1 19, FM1 11	Playa Unión-Barranca Norte	1040±70	Peralta, 2001 quoted in [52]	CENPAT, Puerto Madryn (Argentina)
*Arctocephalus australis*	2	90, 91	Bajada de los pescadores	2197±38	[51]	INCUAPA-UNCPBA, Buenos Aires F.D. (Argentina)
*Otaria flavescens*	2	36, 82	Los Abanicos 1	380±60	[105]	CENPAT, Puerto Madryn (Argentina)
*Otaria flavescens*	9	F1 17, FM1 13–17, FM1 19–21	Playa Unión-Barranca Norte	1040±70	Peralta, 2001 quoted in [52]	CENPAT, Puerto Madryn (Argentina)
*Otaria flavescens*	10	i1(61), i3(43), i4(44), i5(54), i15(C1), i18(89), i23, i24, M11, M13	Lobos*	1290±100	[106]	CENPAT, Puerto Madryn (Argentina)
*Otaria flavescens*	2	FSM - SRH Mont II OF costilla, OBS 13	Faro San Matías- Sondeo 6	1380±80	[51]	INCUAPA-UNCPBA, Buenos Aires F.D. (Argentina)
*Otaria flavescens*	1	OBS 137	Bajada de los pescadores	2197±38	[51]	INCUAPA-UNCPBA, Buenos Aires F.D. (Argentina)
*Otaria flavescens*	2	FSM - SRH Mont I OF cost px med, OBS 4	Faro San Matías, Sondeo 2	2910±90	[51]	INCUAPA-UNCPBA, Buenos Aires F.D. (Argentina)
*Spheniscus magellanicus*	4	7,72, 80, 82	Bajada de los pescadores	2197±38	[51]	INCUAPA-UNCPBA, Buenos Aires F.D. (Argentina)
*Spheniscus magellanicus*	3	OBS 26, FSM-S2N2 Obs 21, FSM-S2N3 Obs26-tibia derecha.	Faro San Matías, Sondeo 2	2910±90	[51]	INCUAPA-UNCPBA, Buenos Aires F.D. (Argentina)

*Lobos is a paleontological site, since here sea lions died naturally and massively due to a land slide.

**Table 3:** Archaeological samples from southern Patagonia.

Species	N	Laboratory ID	Archaeological site	Age (^14^C yr BP)	Reference	Repository information
**Herbivores**						
*Mytilus edulis*	5	A17-cM1, M2, M3, M4, M5	Tunel VII	100±45	Piana et al., 1992 quoted in [107]	CENPAT, Puerto Madryn (Argentina)
*Mytilus edulis*	3	A15-cM1, M2, M3	Shamakush X, Capa E	500±100	Orquera and Piana, 1999 quoted in [50]	CENPAT, Puerto Madryn (Argentina)
*Mytilus edulis*	5	A19-cM1, M2, M3, M4, M5	Imiwaia I (M/K)	5940±50, 5750±170, 5840±45, 5710±50	Piana et al., 1992 quoted in [107]	CENPAT, Puerto Madryn (Argentina)
*Nacella magellanica*	5	A17-cL1, L2, L3, L4, L5	Tunel VII	100±45	Piana et al., 1992 quoted in [107]	CENPAT, Puerto Madryn (Argentina)
*Nacella magellanica*	2	A15-cL2, L4	Shamakush X, Capa E	500±100	Orquera and Piana, 1999 quoted in [50]	CENPAT, Puerto Madryn (Argentina)
*Nacella magellanica*	5	A19-cL1, L2, L3, L4, L5	Imiwaia I (M/K)	5940±50, 5750±170, 5840±45, 5710±50	Piana et al., 1992 quoted in [107]	CENPAT, Puerto Madryn (Argentina)
**Top predators**						
*Arctocephalus australis*	13	44331, 155288, 150329, 152253, 152439, 151607, 154656, 151575, 151912, 154284, 153887, 55456, 155447	Tunel VII, layer B	100±45	Piana et al., 1992 quoted in [107]	CADIC, Ushuaia (Argentina)
*Arctocephalus australis*	1	CV6 4/-45–50 cm	CV6 4/-45-50 cm	1190±60	[108]	INCUAPA-UNCPBA/IMHICIHU, Buenos Aires F. D. (Argentina)
*Arctocephalus australis*	1	CdN2-0072	Cueva del Negro-cuadrícula 1 Nivel 2	1730±80	[109]	CENPAT, Puerto Madryn (Argentina)
*Arctocephalus australis*	3	37295, 37456, 37340	Tunel I, Capa X/α	2660±100,	[110]	CADIC, Ushuaia (Argentina)
				2690±80, 3030±90		
*Arctocephalus australis*	10	194047, 43247, 174498, 193261, 67319, 66397, 202083, 186854, 65989, 202401	Tunel I, Capa D	5000–4300	[110]	CADIC, Ushuaia (Argentina)
*Arctocephalus australis*	20	217933, 215241, 223614, 215940/215933, 53580, 68445, 64460, 213370, 215074, 58630, 190846, 63330, 213732, 52463, 226119, 69639, 189603, 216713, 212616/212653, 224151	Tunel I, Capa D/E	6400–5900	[110]	CADIC, Ushuaia (Argentina)
*Otaria flavescens*	2	43418, 154286	Tunel VII	100±45	Piana et al., 1992 quoted in [107]	CADIC, Ushuaia (Argentina)
*Otaria flavescens*	10	OF 2a, OF 2b, OF 3, OF 9, OF 12, OF 14, OF 15, OF 5, OF 7, OF 10	Kaiyawoteha III, Capa K	580±45	[111]	CADIC, Ushuaia (Argentina)
*Otaria flavescens*	1	CV6 4/-60–65 cm	CV6 4/-60-65 cm	1190±60	[108]	INCUAPA-UNCPBA/IMHICIHU, Buenos Aires F. D. (Argentina)
*Otaria flavescens*	5	CV20 OF1, CV20 OF3, CV20 OF4, CV20 OF6, CV20 OF7	CV20	1256±50	[112]	CONICET, Río Gallegos/CONICET-IMHICIHU, Buenos Aires (Argentina)
*Otaria flavescens*	9	30459, 33459, 33551, 33571, 33717, 34177, 34544, 34751, 33458	Tunel I, Capa D	5000–4300	[110]	CADIC, Ushuaia (Argentina)
*Spheniscus magellanus*	2	10030, 10100	Shamakush X, Capa E	500±100	Orquera and Piana, 1999 quoted in [50]	CADIC, Ushuaia (Argentina)
*Spheniscus magellanus*	3	pingüino 4a, pingüino 4b, pingüino 6,	Kaiyawoteha III, Capa K	580±45	[111]	CADIC, Ushuaia (Argentina)
*Spheniscus magellanus*	4	12433, 12268, 10116, 10115	Mischiuen I, Capa C sup	890±90	Piana et al., 2004 quoted in [113]	CADIC, Ushuaia (Argentina)
*Spheniscus magellanus*	1	9255	Mischiuen I, Capa C inf	1060±85	Piana et al., 2004 quoted in [113]	CADIC, Ushuaia (Argentina)
*Spheniscus magellanus*	2	19098, 19264	Shamakush I, Capa D	1220±110, 940±110	[114]	CADIC, Ushuaia (Argentina)
*Spheniscus magellanus*	1	10122	Shamakush I, Capa C	1000 (ca.)	[114]	CADIC, Ushuaia (Argentina)
*Spheniscus magellanus*	4	3761, 4179, 3522, 3641	Mischiuen I, Capa F	4890±210, 4430±180	Piana et al., 2004 quoted in [113]	CADIC, Ushuaia (Argentina)
*Spheniscus magellanus*	3	1925, 26006, 27597	Imiwaia I (M/K)	5940±50, 5750±170, 5840±45, 5710±50	Piana et al., 1992 quoted in [107]	CADIC, Ushuaia (Argentina)

### Study site and sample collection

**Figure 1:**
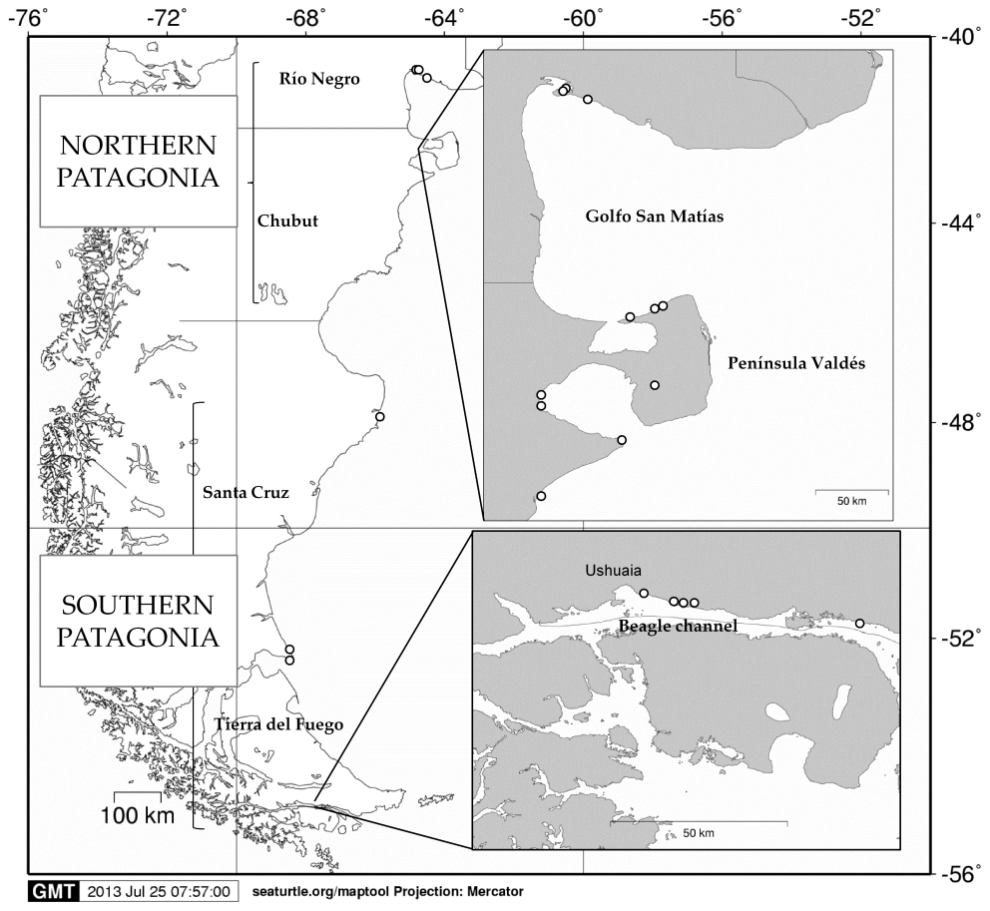
Study areas (central-northern Patagonia and southern Patagonia) and sampling approximate zones. Empty dots represent archaeological sites.

Bones and shells are commonly used in paleontological and archaeological isotopic studies because they contain organic remains and they are often abundant in archaeological deposits as well as in historic museum collections [24], [58], [59], [60]. Furthermore, the proteins they contain integrate the diet over several years [61], [62], [63]. Accordingly, we determined the ratios of stable isotopes of carbon and nitrogen in the organic matrix from the shell of modern and archaeological intertidal mussels (*Aulacomya atra atra* and *Mytilus edulis*) and limpets (*Nacella magellanica*), and in modern and archaeological bone tissue from South American sea lions, South American fur seals and Magellanic penguins. Mussels and limpets were used to characterise the trophic level 1 (herbivores), which allows us to interpret whether shifts in the predators are more likely linked to prey switching or a change in the isotopic baseline. Both modern and archaeological samples were collected in two areas of the South-east coast of South America (Figure 1): central-northern Patagonia (Río Negro and Chubut provinces) and southern Patagonia (Santa Cruz and Tierra del Fuego provinces). These two areas differ in oceanographic, biogeographic and anthropogenic features, with central-northern Patagonia being less productive and more affected by anthropogenic impacts (industrial fishing) than southern Patagonia [64], [65].

Nasal turbinates from modern marine mammal were sampled from specimens at the scientific collections of the *Centro Nacional Patagonico* (Puerto Madryn, Argentina) and *Museo Acatushún* (Ushuaia, Argentina) and analysed. Modern penguin bones from adult penguins found dead at breeding colonies distributed along central-northern Patagonia and southern Patagonia were also analysed. Modern mollusc samples were collected from December 2009 to February 2010 at three sites along the coastline of Argentina: two in the Río Negro province (central-northern Patagonia) and one in the Tierra del Fuego province (southern Patagonia) (Figure 1, Table 1).

Sex is often difficult to assess when only fragmented individuals are recovered from the zooarchaelogical record, despite being a major determiner in the foraging habits of fur seals, sea lions and penguins [47], [66], [67], [68], [69]. Accordingly, a sensitivity analysis was run using modern samples from the Río de la Plata region (between southern Brazil and Buenos Aires province) to assess the sensitivity of food web reconstruction using SIBER (see Data analysis). Blue mussels (*Mytilus edulis*) were collected in the Río de la Plata region to represent ribbed mussel (*Aulacomya atra atra*), and the limpet *Siphonaria lessoni* to represent *Nacella magellanica* (Table 1).

Zooarchaeological bones (generally humeri, mandibles, coxal bones, ribs and femurs) and shell samples recovered from shell middens come from previous fieldwork carried out by Julieta Gómez Otero, Florencia Borrella, Martín Serrán and Lorena Peralta in Golfo de San Matías and Península Valdés (central-northern Patagonia), Eduardo Moreno in Santa Cruz (southern Patagonia) and Ernesto Piana, Luis Orquera, Angie Tivoli and Francisco Zangrando in the Beagle Channel (Tierra del Fuego) (Figure 1, Tables 2 and 3). The samples were dated in different laboratories and using different methods; in particular, in central-northern Patagonia, almost all dated samples were marine shells instead of charcoal. As CO_2_ diffuses slowly from the atmosphere to the ocean, changes in the abundance of ^14^C in sea water are delayed in comparison to changes in the atmosphere [70]. Such a lag is known as the reservoir effect and is the responsible for the difference between the radiocarbonic age of coal and shells from the same archaeological level. Reservoir effect data for the central-northern Patagonia region are emerging only recently [71], and they suggest a relatively small difference between marine and terrestrial ages on the basis of a few samples. Although the use of a correction for reservoir effect would be preferable [52], we refer to the conventional, uncalibrated radiocarbon dates for all the sites. This shortcoming should not be of great importance in these types of studies for which precise dates are not required, but large time intervals should suffice.

All samples were stored in a freezer at −20°C until analysis.

### Stable isotope analysis

Once in the laboratory, bone and shell samples were thawed and dried in an oven at 50°C, and ground to a fine powder with a mortar and pestle. Shell samples were pre-polished with sandpaper and with a diamond wheel drill to remove impurities. They were subsequently rinsed with distilled water and lipids were extracted in all samples with a chloroform/methanol (2∶1) solution [72].

Since both bone and shell contain high concentrations of inorganic carbon, which may bias δ^13^C values [73], they were divided into two aliquots. The first was decarbonised by soaking in 0.5 N (bone) or 1 N (shell) hydrochloric acid (HCl) until no more CO_2_ was released [74]. Since the HCl treatment adversely affects δ^15^N values [75], the second aliquot was not treated with HCl and used for δ^15^N determination.

Dried powdered samples and secondary reference standards were combusted at 900°C, and analysed in a continuous flow isotope ratio mass spectrometer (Flash 1112 IRMS Delta C Series EA Thermo Finnigan). For the shell samples, a CO_2_ absorbent for elemental analyses (CaO/NaOH) was employed to avoid the saturation of the spectrometer during the analysis of the non-acid washed samples, constituted by 90% CaCO_3_. Stable isotope abundance is expressed in standard δ notation relative to carbonate Pee Dee Belemnite and atmospheric nitrogen. Analyses were performed at the Science and Technology Centre (CCiT) of the University of Barcelona.

### Data analysis

Limpets and mussels were analysed together under the “herbivore” category, representing, in the same functional group, both benthic (limpets) and pelagic (mussels) compartments [76]. Each top predator species was analysed separately. Archaeological data were grouped into broad time intervals to enable quantitative reconstruction of food webs. Consequently in central-northern Patagonia all the archaeological samples were pooled together under a single time interval, from 5200±70 yr ^14^C BP until 380±60 yr ^14^C BP. This period is entitled the “Pre-contact period” since it predates the arrival of European settlers, opposed to the term post-contact used in archaeology [51], [53], [77]. Ancient samples from southern Patagonia were split in two different periods. The early aboriginal period (EAP) ranging from 6000 to 1100 years ago was characterised by very high oceanic primary productivity [78] and the prevalence of pinnipeds in the economy of hunter-gatherers [50]. Conversely, the late aboriginal period (LAP), ranging from 1000 to 100 years ago, was characterised by a much lower oceanic primary productivity [78] and a lower reliance of the hunter-gatherers economy on pinnipeds. Modern data were analysed separately, so five different food webs were analysed: two in central-northern Patagonia (one archaeological and one modern) and three in southern Patagonia (two archaeological and one modern).

SIBER (Stable Isotope Bayesian Ellipses in R) [79] was used to compute Bayesian Layman's metrics that summarised food web structures in each region and epoch. Only five of the six measures proposed by Layman et al. (2007) [27] were calculated. The δ^15^N, δ^13^C ranges (NR and CR, respectively) and the mean distance to centroid (CD) are measures of the total extent of spacing within the δ^13^C–δ^15^N bi-plot space and gives a measure of community niche width that is not particularly sensitive to sample size. Nitrogen range (NR) is the representation of the vertical structure of the web; carbon range (CR) gives us an idea of the trophic diversity at the base of the web, while the mean distance to centroid provides a measure of the average degree of trophic diversity within a food web [27]. Mean nearest neighbour distance (MNND) and the standard deviation of nearest neighbour distance (SDNND) reflect the relative position of species to each other within the niche space and can be used to estimate the extent of trophic redundancy.

Furthermore, the overlap (‰) among top predators' standard ellipses corrected for small sample size (SEA_C_) was calculated in order to analyse resource partitioning among them over time. Comparison of metrics was based on 95% credibility intervals. All codes for SIBER analyses are contained in the package SIAR [80], [81]. Finally, SIBER Bayesian ellipse areas (SEA_B_) were calculated for the three top predators to measure their isotopic niche width. This approach is similar to a bootstrap, assigning measures of uncertainty, based on Markov-Chain Monte Carlo (MCMC) simulation to construct parameters of ellipses.

In addition, a simulation was performed to test the sensitivity of SIBER metrics to biases in the sex ratio of the top predators. A similar data set from Río de la Plata was used for this analysis (see Table S1). Three different scenarios were simulated: one where only females were included, one where only males were included and a third situation with a balanced sex ratio. These three simulated food webs should represent the three hypothetical and extreme situations emerging from the analysis of the zooarchaeological record, where the sex of the top predators was usually unknown.

## Results

**Figure 2:**
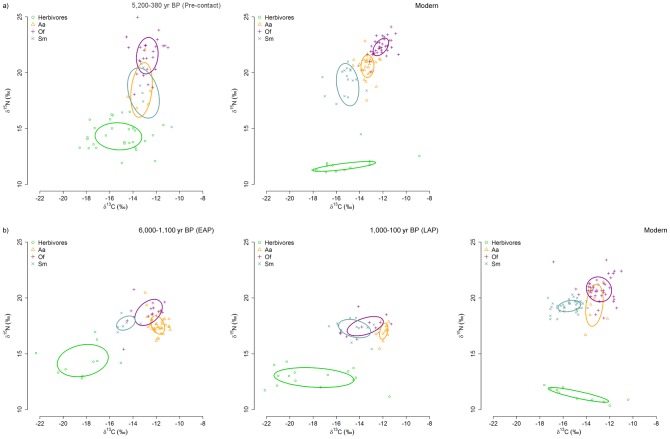
Isotopic niches/resource use areas of the species/functional groups described in the text and calculated with the standard ellipse areas corrected for small sample size (SEA_C_) over time in the two geographical areas. a) Central-northern Patagonia and b) Southern Patagonia. Herbivores = mussels and limpets; Aa = South American fur seals; Of = South American sea lions; Sm = Magellanic penguins.

The structures of the five food webs analysed are shown in Figure 2. The δ^15^N values of herbivores decreased from past to present (Table 4) both in central-northern Patagonia (Wilcoxon-Mann-Whitney test: W = 3, p<0.001) and in southern Patagonia (ANOVA: F_2,32_ = 24.7, p<0.01). Likewise the δ^13^C values of ancient herbivores from southern Patagonia differed from the modern ones (ANOVA: F_2,32_ = 5.42, p<0.01) but changes were not statistically significant in central-northern Patagonia (t-test: t = −0.09, df = 16.777, p = 0.93). These results demonstrate that the isotopic baseline may change dramatically throughout time and allow us to properly interpret the structure of the ancient food webs.

**Table 4:** Mean and standard deviation of nitrogen and carbon stable-isotope in central-northern and southern Patagonia.

Central-northern Patagonia				δ^15^N (‰)		δ^13^C (‰)	
Group	Tissue	N		Mean (±SD)		Mean (±SD)	
		Pre-contact	Modern	Pre-contact	Modern	Pre-contact	Modern
**Herbivores**	Shell	28	12	14.30 (±1.19)	11.61 (±0.42)	−15.25 (±1.97)	−15.32 (±2.57)
**Top Predators**							
*Arctocephalus australis*	Bone	5	29	18.45 (±2.12)	20.59(±0.99)	−13.26 (±0.80)	−13.32 (±0.54)
*Otaria flavescens*	Bone	26	36	21.52 (±1.60)	22.30 (±0.78)	−12.77 (±0.91)	−12.17 (±0.66)
*Spheniscus magellanicus*	Bone	7	20	18.19(±2.05)	18.93 (±1.85)	−13.10 (±1.25)	−15.03 (±0.94)

Southern Patagonia					δ^15^N (‰)			δ^13^C (‰)		
Group	Tissue	N			Mean (±SD)			Mean (±SD)		
		EAP	LAP	Modern	EAP	LAP	Modern	EAP	LAP	Modern
**Herbivores**	Shell	10	15	10	14.39 (±1.36)	12.87 (±0.86)	11.32 (±0.62)	−18.32 (±2.07)	−17.93 (±3.31)	−14.69 (±2.46)
**Top Predators**										
*Arctocephalus australis*	Bone	35	13	7	17.58 (±0.81)	17.02 (±0.69)	19.34 (±1.72)	−11.86 (±0.58)	−11.92 (±0.34)	−13.31 (±0.69)
*Otaria flavescens*	Bone	15	12	41	18.70 (±1.13)	17.47 (±0.82)	20.76 (±1.10)	−12.66 (±1.13)	−13.52 (±1.48)	−12.92 (±1.07)
*Spheniscus magellanicus*	Bone	7	13	40	17.75 (±0.58)	17.25 (±0.79)	19.27 (±0.51)	−14.61 (±0.74)	−14.43 (±1.33)	−15.46 (±0.96)

The three food webs simulated for the sensitivity analysis did not differ in any of the Layman's metrics (Table 5). The areas of the Bayesian ellipses (SEA_B_) estimated for the top predators did not either differ among scenarios either, except for fur seals, whose area was maximised when sex ratio was balanced. However, large differences were observed in the overlap between the standard ellipses corrected for small sample size (SEA_C_) of fur seals and penguins: it ranged from zero when only male fur seals were considered, to 43.73% (fur seals) and 21.34% (penguins) when only females were included. This indicates that the lack of information on the actual sex ratio of ancient data sets is unlikely to bias the overall structure of the food web. Nonetheless caution is needed when interpreting patterns of niche overlap, as they may be sensitive to the sex ratio of the sample.

**Table 5:** Probability values of Layman's metrics in three simulated model food webs for Río de la Plata region, including only females, males or both sexes in the samples of predators.

	NR	CR	CD	MNND	SDNND
**♀♀**	8.01 (7.00–9.04)	6.03 (4.46–7.64)	3.14 (2.77–3.51)	2.95 (2.50–3.41)	2.43 (1.55–3.35)
**♂♂**	8.32 (7.82–8.82)	6.16 (4.75–7.55)	3.44 (3.13–3.76)	3.23 (2.90–3.55)	2.50 (1.80–3.22)
**♀♂**	8.22 (7.55–8.87)	6.03 (4.57–7.42)	3.25 (2.93–3.57)	3.14 (2.75–3.55)	2.39 (1.69–3.10)

NR  =  nitrogen range; CR  =  carbon range; CD  =  mean distance to centroid; MNND  =  mean nearest neighbour distance; SDNND  =  standard deviation of nearest neighbour distance. For more details see *Data analysis* in Material and Methods.

**Figure 3:**
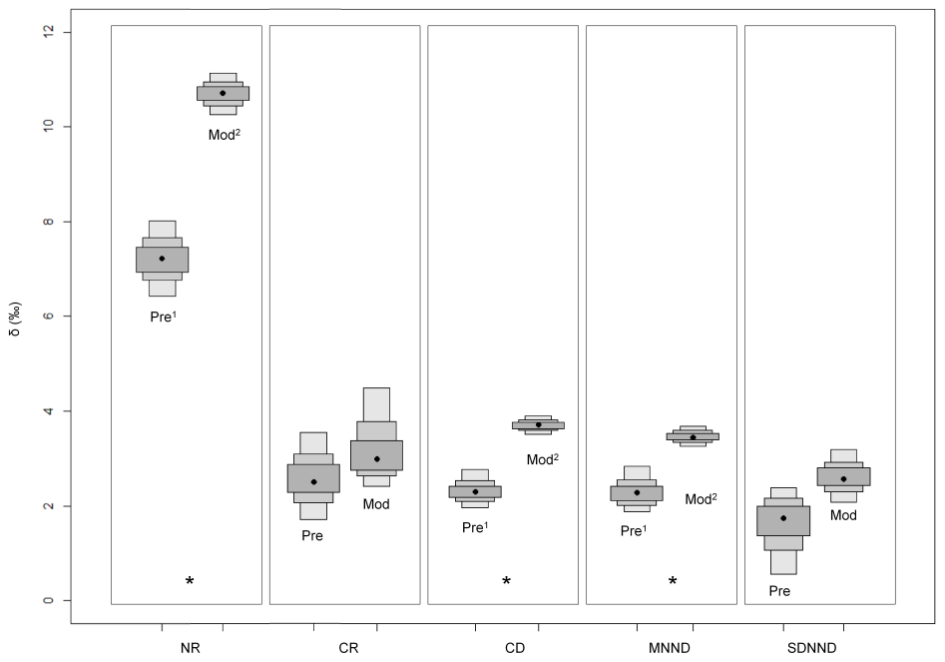
Probability values of Layman's metrics over the time in the central-northern Patagonia. Metrics that present differences over the time are indicated by an asterisk and superscripts. Pre = pre-contact period; Mod =  modern period.

**Figure 4:**
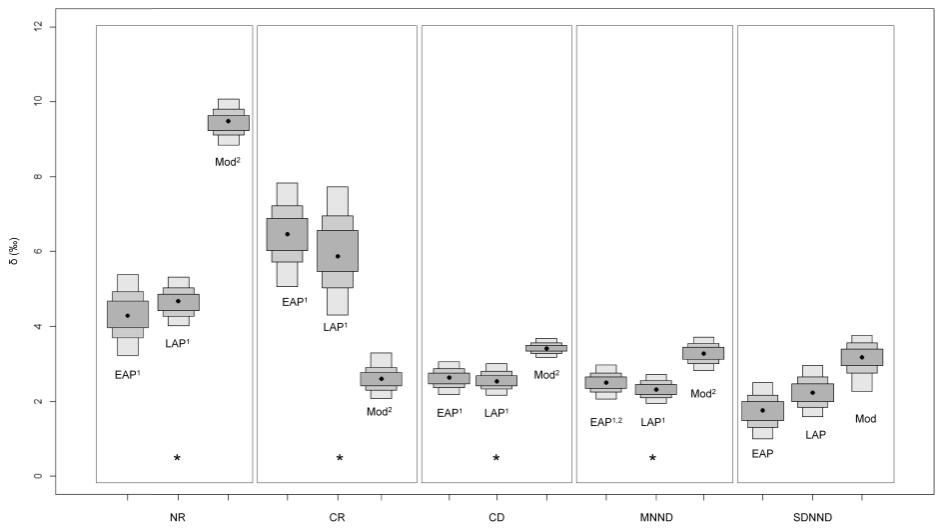
Probability values of Layman's metrics over the time in the southern Patagonia. Metrics that present differences over the time are indicated by an asterisk ad superscripts. Groups sharing the same superscript number (1, 2) are not significantly different. EAP = early aboriginal period; LAP = late aboriginal period; Mod = modern period.

The horizontal structure of the food web (CR) did not vary throughout time in central-northern Patagonia but in southern Patagonia the carbon range is currently smaller than in the two past periods considered (Figure 3 and 4). The nitrogen range (NR), indicative of food chain length, increased from past to present in both regions, and did not differ between ancient food webs from southern Patagonia (Figure 3 and 4). Finally, the food webs from southern Patagonia were shorter than those from central-northern Patagonia during the late Holocene as well as in the present.

CD and MNND also changed throughout time in both regions, being smaller in the past. This indicates an increase in the trophic diversity and a decrease in the trophic redundancy in modern food webs. Regarding the three species of air predators the areas of the standard ellipses did not overlap in the modern δ^13^C–δ^15^N bi-plot space of central-northern Patagonia (Table 6 and Figure 2). Conversely, the topology of the three species within the Pre-contact δ^13^C–δ^15^N bi-plot space of the same region departed dramatically from expectations, as the standard ellipses of all the species overlapped one another, especially those of Magellanic penguins and fur seals, in sharp contrast with the low overlap currently observed (Table 6). The topology of the three species in the δ^13^C–δ^15^N bi-plot space of southern Patagonia also changed over time, as the overlap among the ellipses of the three species was almost zero in the EAP period (except between sea lions and fur seals where it was 15.38%), increased between penguins and sea lions in the LAP period and is currently about 34% between fur seals and sea lions. Interestingly, the SIBER ellipse areas (SEA_B_) calculated for each species (Table 7) exhibited a decreasing trend throughout time in central-northern Patagonia, although it was statistically significant only for sea lions. Conversely in southern Patagonia, the sea lions' ellipse area has been constant over time, whereas that of fur seals showed a trend to increase through time and that of penguins slightly increased from the EAP period to the LAP period to then slightly decreased in the present food web (Table 7).

**Table 6:** Overlap as a percentage of standard ellipses corrected for small samples (SEA_c_) of the three top predators described in the text.

Central-northern Patagonia							
PRE-CONTACT	Aa	Of	Sm	MODERN	Aa	Of	Sm
**Aa**	**1**	11.37	86.30	**Aa**	**1**	0.00	0.00
**Of**	16.42	**1**	5.05	**Of**	0.00	**1**	0.00
**Sm**	64.56	2.62	**1**	**Sm**	0.00	0.00	**1**

Southern Patagonia											
EAP	Aa	Of	Sm	LAP	Aa	Of	Sm	MODERN	Aa	Of	Sm
**Aa**	**1**	15.38	0.00	**Aa**	**1**	0.00	0.00	**Aa**	**1**	34.19	0.00
**Of**	5.60	**1**	0.05	**Of**	0.00	**1**	53.24	**Of**	38.42	**1**	0.00
**Sm**	0.00	0.13	**1**	**Sm**	0.00	58.28	**1**	**Sm**	0.00	0.00	**1**

The tables should be read horizontally, as each number in the cell refers to the percentage of overlap of the area of the group indicated in each row (e.g. 11.37% is the percentage of the ellipses of fur seals that are overlapped with the ellipses of the sea lions, while 16.42% is the percentage of the ellipses of the sea lions that are overlapped with the fur seals). Aa = *Arctocephalus australis*; Of = *Otaria flavescens*; Sm = S*pheniscus magellanicus*.

**Table 7:** Areas of the estimated Bayesian ellipses of the species/functional groups over the time in central-northern and southern Patagonia.

Central-northern Patagonia	Herbivores	Aa	Of	Sm
Pre-contact	7.4 (4.9–10.3)	6.1 (1.9–11.7)	4.7 (3.0–6.6)^1^	7.7 (3.1–13.6)
Modern	3.9 (2.0–6.3)	1.9 (1.3–2.6)	1.6 (1.1–2.1)^2^	5.5 (3.3–8.1)
**Southern Patagonia**				
EAP	8.6 (4.1–14.2)	1.6 (1.1–2.1)	4.0 (2.1–6.1)	2.2 (0.9–3.8)
LAP	9.0 (5.0–13.9)	1.3 (0.7–2.0)	3.9 (2.0–6.2)	3.5 (1.8–5.5)
Modern	4.1 (1.9–6.7)	4.4 (1.8–7.8)	3.8 (2.7–5.0)	1.7 (1.2–2.2)

Values are indicated as the mean and the 95% of credibility interval in parentheses. Metrics that present differences between ages are indicated by superscripts. Aa = *Arctocephalus australis*; Of = *Otaria flavescens*; Sm = S*pheniscus magellanicus*.

All the results of the stable isotopes analyses described in the above are publicly available in Table S1–S3.

## Discussion

The study of the zooarchaeological record using stable isotope analysis certainly offers a window to explore the structure of ancient food webs, but it is not free from problems. Historical changes in the isotopic baseline are a major shortcoming in retrospective studies using stable isotope analysis to assess changes in trophic level and food web structure [57]. Although widely recognised as a confusing factor, previous studies often assumed the temporal stability of the isotopic baseline [82], [83], [84], [85], [86], [87], [88], [89], but the results reported here demostrate that changes can be dramatic. Post [76] suggested the use of filter feeding mussels and surface-grazing snails as proxies for the baselines of pelagic and littoral aquatic food webs respectively and Bailey and colleagues [88] and Casey and Post [57] recognised the potential of mollusc shells to reconstruct the isotopic baseline in retrospective studies, since their organic matrix is encased within mineral crystals and hence, preserved [90]. The present study demonstrates the potential of the method and offers new perspectives using material that is widely available in paleontological and archaeological collections.

Another drawback from retrospective studies is the uneven distribution of specimens across space and time, which forced us to pool samples of disparate radiocarbon age to reconstruct ancient communities. Uncertainty about the gender of most specimens is another setback of the zooarchaeological record, as sexual secondary characters can seldom be observed. The sensitivity analysis conducted here confirmed the robustness of Layman's metrics to changes in sex ratios of penguins, sea lions and fur seals. Conversely, the overlap between the Bayesian ellipses was highly sensitive to the sex ratio of those species and hence caution is needed when interpreting those results.

The overall evidence reported here indicates that modern marine food webs of central-northern and southern Patagonia are longer (NR) than the ancient ones (Figs 2–4). Such a conclusion is in sharp contrast with the idea that human exploitation has shortened food webs because of the preferential removal of top predators [4]. Certainly, increased scarcity of large, marine species may have forced fisheries to target smaller species, but there is no reason why surviving top predators had to experience a similar shift. Marine predators are limited by the size of the prey they can consume and draw their energy from a very limited range of trophic levels, in contrast with fisheries [91]. Furthermore, it should be noted that the impact of sealing on the populations of fur seals and sea lions was much larger [39], [40] than that of fishing on the populations of hake, squid and anchovies [20].

Surviving sea lions and fur seals off Argentina have certainly been under carrying capacity during the second half of the 20^th^ century, when the population was less than 10% of the original numbers [39]. This reduced intraspecific competition and led to a major dietary shift in favour of benthic, larger prey with a higher trophic level [24]. Only recently, as the population of sea lions is rebuilding and approaching carrying capacity, sea lions experience resource limitation again and intraspecific competition forces them to increase the consumption of smaller and less profitable prey [92]. Information about the historical dietary changes of South American fur seals is restricted to the last three decades in Río de la Plata and reveals no major dietary changes [93]. However, the population was dramatically reduced during the first half of the 20^th^ century and evidence from other fur seal species in the South-western Atlantic Ocean [94] suggest that this group of smaller and more pelagic pinnipeds also forage at a higher trophic level after severe population declines.

Trophic diversity, measured as the mean distance to the centroid in the isotopic space (CD) also increases in the present food webs, while redundancy (MNND) decreases thus revealing a higher trophic overlap in the past (Figs 2–4). This was confirmed by the isotopic niche overlap of the three air-breathing top predators, although they differed dramatically in body size and mouth diameter (Table 6). A similar scenario has been reported for waters off Peru, where sea lions, fur seals and penguins primarily rely on the large population of anchoveta *Engraulis ringens*, supported by intense oceanic productivity [95], [96], [97]. Similarly, a high trophic overlap has often been reported for other wasp-waisted ecosystems in upwelling regions [98]. Currently, a wasp-waist ecosystem supported by amphipods exists off southern Patagonia [99], but amphipods are not consumed directly by fur seals and sea lions. Perhaps a similar wasp-waisted ecosystem supported by small schooling fish might have existed off Patagonia during the Holocene, where primary productivity was usually higher than now [78] and hence it could have supported a larger population of small schooling fish and squids.

However, there are at least two reasons to believe that this is not the explanation for the high levels of overlap and redundancy of the ancient food webs reported here. Firstly, the large population of fur seals inhabiting the highly productive Río de la Plata rely heavily on anchovies and squids [93], but the much smaller sea lion population consumes primarily demersal fishes [100]. Secondly, marine productivity during the late aboriginal period off southern Patagonia was much lower than during the early aboriginal period and closer to actual productivity levels [78], but the structure of the trophic web was indistinguishable from that observed in the same region during the early aboriginal period. Thus, a direct link between productivity, predator diet and the structure of trophic overlap is probably unclear.

Empirical evidence has indicated that marine environments with lower annual temperature variability have smaller predator-prey mass ratios and, consequently, longer food chains [101]. It can be argued that changes in sea water temperature throughout the second half of the Holocene may explain the difference observed between the ancient and modern marine food webs in the South-western Atlantic. However, available evidence rules out major changes in sea surface temperature during the considered time span [51], [55], [102]. Furthermore, there is an inverse relationship between the length of the current food webs and the seasonal variability in the sea surface temperature in the South-western Atlantic [103], a pattern opposite to that reported by Jennings and Warr (2003)[101]. Finally, the difference in the length of the food chain observed between the two areas in both periods is smaller than the difference between periods in both areas.

Thus, human disturbance stands as the most likely reason for the differences in the structure of the food web reported here. Even so, human disturbance is not the only factor explaining the length of marine food webs, as those from southern Patagonia were always shorter and more overlapping than the central-northern Patagonia ones. This might be a consequence of the latitudinal decrease in species diversity reported in the South-western Atlantic [64], [104] and was also probably the reason in the late Holocene, since it is unlikely that the impact of the hunter-gatherers inhabiting central-northern Patagonia on marine resources was larger than that of the hunter-gatherers inhabiting southern Patagonia [51], [52], [53].

In conclusion, this study strongly support the hypothesis that selective exploitation of marine ecosystems, targeting primarily top predators, leads to longer and less overlapping food webs, if top predators are not extinct but survive well below carrying capacity. The situation might be different if human exploitation targeted primarily intermediate trophic levels as a result of the legal protection of top predators.

Furthermore, this study demonstrates the necessity to reconstruct the isotopic baseline in retrospective studies and how this can be achieved by analysing the organic matter encased into the shell of molluscs.

## Supporting Information

Table S1 **Modern nitrogen and carbon stable-isotope ratios in Río de la Plata and adjoining areas.** These data have been used to perform the sensitivity test for the method.
(DOCX)

Table S2 **Modern and archaeological nitrogen and carbon stable-isotope ratios in central-northern Patagonia.** Table shows the list of samples, grouped according to their historical period (modern and pre-contact), and δ^13^C and δ^15^N values.
(DOCX)

Table S3 **Modern and archaeological nitrogen and carbon stable-isotope ratios in southern Patagonia.** Table shows the list of samples, grouped according to their historical period (modern, LAP and EAP), and δ^13^C and δ^15^N values.
(DOCX)
