# Supplementary material for: Longer and Less Overlapping Food Webs in Anthropogenically Disturbed Marine Ecosystems: Confirmations from the Past
Source: PLoS One. 2014 Jul 30;9(7):e103132. doi: 10.1371/journal.pone.0103132 (PMC4116168; doi:10.1371/journal.pone.0103132)
Supplement: Table S1 — Modern nitrogen and carbon stable-isotope ratios in Río de la Plata and adjoining areas. These data have been used to perform the sensitivity test for the method. (DOCX) [file pone.0103132.s001.docx]

| **Sample** | **Specie/Group** | **Sex** | **δ^13^C** | **δ^15^N** |
| --- | --- | --- | --- | --- |
| RP-H1 | Herbivore | NA | -17.79 | 12.31 |
| RP-H2 | Herbivore | NA | -16.15 | 11.99 |
| RP-H3 | Herbivore | NA | -17.00 | 11.85 |
| RP-H4 | Herbivore | NA | -16.73 | 12.05 |
| RP-H5 | Herbivore | NA | -17.16 | 11.91 |
| RP-H6 | Herbivore | NA | -17.76 | 12.43 |
| RP-H7 | Herbivore | NA | -17.46 | 13.62 |
| RP-H8 | Herbivore | NA | -22.50 | 12.29 |
| RP-H9 | Herbivore | NA | -16.78 | 12.92 |
| RP-H10 | Herbivore | NA | -21.17 | 12.32 |
| RP-Aa1 | *A. australis* | female | -13.81 | 19.26 |
| RP-Aa2 | *A. australis* | female | -12.80 | 20.05 |
| RP-Aa3 | *A. australis* | female | -13.84 | 17.50 |
| RP-Aa4 | *A. australis* | female | -14.04 | 18.15 |
| RP-Aa5 | *A. australis* | female | -13.44 | 19.14 |
| RP-Aa6 | *A. australis* | male | -16.62 | 20.53 |
| RP-Aa7 | *A. australis* | male | -15.60 | 20.50 |
| RP-Aa8 | *A. australis* | male | -12.87 | 20.52 |
| RP-Aa9 | *A. australis* | male | -13.31 | 20.80 |
| RP-Aa10 | *A. australis* | male | -13.09 | 20.43 |
| RP-Aa11 | *A. australis* | male | -13.11 | 20.93 |
| RP-Aa12 | *A. australis* | male | -13.87 | 20.53 |
| RP-Aa13 | *A. australis* | male | -13.34 | 20.81 |
| RP-Aa14 | *A. australis* | male | -12.97 | 20.07 |
| RP-Aa15 | *A. australis* | male | -14.51 | 19.29 |
| RP-Aa16 | *A. australis* | male | -14.22 | 19.18 |
| RP-Aa17 | *A. australis* | male | -13.34 | 21.23 |
| RP-Aa18 | *A. australis* | male | -14.32 | 22.73 |
| RP-Aa19 | *A. australis* | male | -13.40 | 20.74 |
| RP-Aa20 | *A. australis* | male | -12.58 | 20.64 |
| RP-Aa21 | *A. australis* | male | -12.78 | 20.95 |
| RP-Aa22 | *A. australis* | male | -12.64 | 20.63 |
| RP-Aa23 | *A. australis* | male | -13.25 | 19.46 |
| RP-Aa24 | *A. australis* | male | -13.69 | 21.50 |
| RP-Aa25 | *A. australis* | male | -12.74 | 20.73 |
| RP-Aa26 | *A. australis* | male | -13.04 | 19.88 |
| RP-Aa27 | *A. australis* | male | -12.62 | 19.76 |
| RP-Aa28 | *A. australis* | male | -13.50 | 21.11 |
| RP-Aa29 | *A. australis* | male | -13.19 | 20.55 |
| RP-Aa30 | *A. australis* | male | -14.15 | 21.19 |
| RP-Aa31 | *A. australis* | male | -14.33 | 21.00 |
| RP-Aa32 | *A. australis* | male | -14.44 | 19.82 |
| RP-Aa33 | *A. australis* | male | -12.90 | 20.18 |
| RP-Aa34 | *A. australis* | male | -15.19 | 20.17 |
| RP-Aa35 | *A. australis* | male | -14.08 | 20.23 |
| RP-Aa36 | *A. australis* | male | -14.27 | 19.80 |
| RP-Aa37 | *A. australis* | male | -13.63 | 20.08 |
| RP-Aa38 | *A. australis* | male | -13.55 | 20.58 |
| RP-Aa39 | *A. australis* | male | -14.09 | 20.41 |
| RP-Aa40 | *A. australis* | male | -13.93 | 20.46 |
| RP-Aa41 | *A. australis* | male | -12.78 | 20.72 |
| RP-Aa42 | *A. australis* | male | -13.84 | 20.91 |
| RP-Aa43 | *A. australis* | male | -13.71 | 20.98 |
| RP-Aa44 | *A. australis* | male | -14.32 | 20.37 |
| RP-Aa45 | *A. australis* | male | -12.87 | 20.57 |
| RP-Aa46 | *A. australis* | male | -13.10 | 19.68 |
| RP-Aa47 | *A. australis* | male | -13.57 | 21.16 |
| RP-Aa48 | *A. australis* | male | -13.94 | 21.00 |
| RP-Aa49 | *A. australis* | male | -12.84 | 21.54 |
| RP-Aa50 | *A. australis* | male | -13.24 | 20.59 |
| RP-Aa51 | *A. australis* | male | -12.84 | 20.53 |
| RP-Aa52 | *A. australis* | male | -13.23 | 21.00 |
| RP-Aa53 | *A. australis* | male | -13.53 | 20.81 |
| RP-Aa54 | *A. australis* | male | -12.50 | 21.17 |
| RP-Aa55 | *A. australis* | male | -13.13 | 21.32 |
| RP-Aa56 | *A. australis* | male | -14.16 | 19.60 |
| RP-Aa57 | *A. australis* | male | -13.28 | 20.56 |
| RP-Aa58 | *A. australis* | male | -13.23 | 20.47 |
| RP-Aa59 | *A. australis* | male | -13.20 | 20.72 |
| RP-Of1 | *O. flavescens* | female | -12.23 | 20.71 |
| RP-Of2 | *O. flavescens* | female | -11.68 | 20.42 |
| RP-Of3 | *O. flavescens* | female | -11.82 | 20.22 |
| RP-Of4 | *O. flavescens* | female | -12.33 | 20.13 |
| RP-Of5 | *O. flavescens* | male | -12.01 | 20.67 |
| RP-Of6 | *O. flavescens* | male | -12.30 | 20.80 |
| RP-Of7 | *O. flavescens* | male | -11.45 | 21.23 |
| RP-Of8 | *O. flavescens* | male | -12.40 | 20.44 |
| RP-Of9 | *O. flavescens* | male | -12.20 | 21.44 |
| RP-Of10 | *O. flavescens* | male | -11.04 | 20.09 |
| RP-Of11 | *O. flavescens* | male | -13.73 | 20.58 |
| RP-Of12 | *O. flavescens* | male | -11.85 | 20.92 |
| RP-Of13 | *O. flavescens* | male | -11.45 | 21.23 |
| RP-Of14 | *O. flavescens* | male | -12.73 | 20.45 |
| RP-Of15 | *O. flavescens* | male | -11.79 | 20.92 |
| RP-Of16 | *O. flavescens* | male | -11.41 | 20.06 |
| RP-Of17 | *O. flavescens* | male | -10.81 | 20.45 |
| RP-Of18 | *O. flavescens* | male | -12.11 | 20.78 |
| RP-Of19 | *O. flavescens* | male | -11.23 | 19.80 |
| RP-Sm1 | *S. magellanicus* | female | -14.30 | 19.70 |
| RP-Sm2 | *S. magellanicus* | female | -13.00 | 17.00 |
| RP-Sm3 | *S. magellanicus* | female | -14.90 | 15.00 |
| RP-Sm4 | *S. magellanicus* | female | -14.10 | 20.10 |
| RP-Sm5 | *S. magellanicus* | female | -13.10 | 17.00 |
| RP-Sm6 | *S. magellanicus* | female | -15.90 | 17.40 |
| RP-Sm7 | *S. magellanicus* | female | -14.90 | 17.30 |
| RP-Sm8 | *S. magellanicus* | female | -15.20 | 17.10 |
| RP-Sm9 | *S. magellanicus* | female | -15.40 | 17.30 |
| RP-Sm10 | *S. magellanicus* | female | -14.50 | 20.00 |
| RP-Sm11 | *S. magellanicus* | male | -14.10 | 16.00 |
| RP-Sm12 | *S. magellanicus* | male | -13.90 | 20.50 |
| RP-Sm13 | *S. magellanicus* | male | -11.30 | 14.50 |
| RP-Sm14 | *S. magellanicus* | male | -14.20 | 18.30 |
| RP-Sm15 | *S. magellanicus* | male | -14.00 | 20.20 |
| RP-Sm16 | *S. magellanicus* | male | -15.30 | 17.40 |
| RP-Sm17 | *S. magellanicus* | male | -15.80 | 17.20 |
| RP-Sm18 | *S. magellanicus* | male | -14.80 | 17.40 |
| RP-Sm19 | *S. magellanicus* | male | -14.60 | 19.20 |
| RP-Sm20 | *S. magellanicus* | male | -14.10 | 19.40 |
