# Supplementary material for: Longer and Less Overlapping Food Webs in Anthropogenically Disturbed Marine Ecosystems: Confirmations from the Past
Source: PLoS One. 2014 Jul 30;9(7):e103132. doi: 10.1371/journal.pone.0103132 (PMC4116168; doi:10.1371/journal.pone.0103132)
Supplement: Table S2 — Modern and archaeological nitrogen and carbon stable-isotope ratios in central-northern Patagonia. Table shows the list of samples, grouped according to their historical period (modern and pre-contact), and δ13C and δ15N values. (DOCX) [file pone.0103132.s002.docx]

| **Sample** | **Species/Group** | **Period** | **δ^13^C** | **δ^15^N** |
| --- | --- | --- | --- | --- |
| NP-H1 | Herbivore | modern | -16.80 | 11.70 |
| NP-H2 | Herbivore | modern | -14.80 | 11.50 |
| NP-H3 | Herbivore | modern | -16.90 | 11.10 |
| NP-H4 | Herbivore | modern | -16.80 | 11.90 |
| NP-H5 | Herbivore | modern | -15.30 | 11.30 |
| NP-H6 | Herbivore | modern | -18.05 | 11.30 |
| NP-H7 | Herbivore | modern | -17.72 | 11.30 |
| NP-H8 | Herbivore | modern | -8.88 | 12.54 |
| NP-H9 | Herbivore | modern | -13.13 | 11.76 |
| NP-H10 | Herbivore | modern | -16.03 | 11.16 |
| NP-H11 | Herbivore | modern | -13.14 | 12.06 |
| NP-H12 | Herbivore | modern | -16.33 | 11.71 |
| NP-Aa1 | *A. australis* | modern | -13.55 | 22.83 |
| NP-Aa2 | *A. australis* | modern | -12.30 | 21.24 |
| NP-Aa3 | *A. australis* | modern | -13.12 | 21.28 |
| NP-Aa4 | *A. australis* | modern | -13.40 | 21.55 |
| NP-Aa5 | *A. australis* | modern | -13.25 | 20.49 |
| NP-Aa6 | *A. australis* | modern | -13.46 | 19.21 |
| NP-Aa7 | *A. australis* | modern | -13.12 | 20.03 |
| NP-Aa8 | *A. australis* | modern | -13.45 | 20.97 |
| NP-Aa9 | *A. australis* | modern | -13.63 | 21.14 |
| NP-Aa10 | *A. australis* | modern | -12.57 | 18.76 |
| NP-Aa11 | *A. australis* | modern | -14.01 | 20.75 |
| NP-Aa12 | *A. australis* | modern | -13.85 | 20.25 |
| NP-Aa13 | *A. australis* | modern | -13.72 | 20.22 |
| NP-Aa14 | *A. australis* | modern | -13.39 | 17.52 |
| NP-Aa15 | *A. australis* | modern | -13.39 | 21.01 |
| NP-Aa16 | *A. australis* | modern | -13.27 | 19.88 |
| NP-Aa17 | *A. australis* | modern | -13.06 | 21.07 |
| NP-Aa18 | *A. australis* | modern | -13.59 | 20.23 |
| NP-Aa19 | *A. australis* | modern | -12.90 | 20.15 |
| NP-Aa20 | *A. australis* | modern | -12.62 | 20.94 |
| NP-Aa21 | *A. australis* | modern | -14.57 | 20.63 |
| NP-Aa22 | *A. australis* | modern | -14.51 | 21.16 |
| NP-Aa23 | *A. australis* | modern | -12.79 | 21.63 |
| NP-Aa24 | *A. australis* | modern | -13.38 | 21.17 |
| NP-Aa25 | *A. australis* | modern | -13.62 | 19.37 |
| NP-Aa26 | *A. australis* | modern | -13.52 | 20.53 |
| NP-Aa27 | *A. australis* | modern | -13.16 | 20.78 |
| NP-Aa28 | *A. australis* | modern | -12.96 | 21.02 |
| NP-Aa29 | *A. australis* | modern | -12.23 | 21.17 |
| NP-Of1 | *O. flavescens* | modern | -12.79 | 21.69 |
| NP-Of2 | *O. flavescens* | modern | -11.90 | 23.00 |
| NP-Of3 | *O. flavescens* | modern | -11.60 | 22.00 |
| NP-Of4 | *O. flavescens* | modern | -11.30 | 24.10 |
| NP-Of5 | *O. flavescens* | modern | -12.10 | 21.80 |
| NP-Of6 | *O. flavescens* | modern | -11.90 | 23.20 |
| NP-Of7 | *O. flavescens* | modern | -11.60 | 23.40 |
| NP-Of8 | *O. flavescens* | modern | -12.51 | 22.68 |
| NP-Of9 | *O. flavescens* | modern | -11.29 | 22.90 |
| NP-Of10 | *O. flavescens* | modern | -11.25 | 22.27 |
| NP-Of11 | *O. flavescens* | modern | -12.85 | 21.86 |
| NP-Of12 | *O. flavescens* | modern | -12.30 | 22.70 |
| NP-Of13 | *O. flavescens* | modern | -11.40 | 22.90 |
| NP-Of14 | *O. flavescens* | modern | -11.70 | 21.50 |
| NP-Of15 | *O. flavescens* | modern | -12.13 | 22.03 |
| NP-Of16 | *O. flavescens* | modern | -10.66 | 21.62 |
| NP-Of17 | *O. flavescens* | modern | -10.90 | 23.50 |
| NP-Of18 | *O. flavescens* | modern | -12.75 | 21.69 |
| NP-Of19 | *O. flavescens* | modern | -12.26 | 22.21 |
| NP-Of20 | *O. flavescens* | modern | -11.64 | 22.42 |
| NP-Of21 | *O. flavescens* | modern | -12.35 | 22.15 |
| NP-Of22 | *O. flavescens* | modern | -13.04 | 21.91 |
| NP-Of23 | *O. flavescens* | modern | -12.57 | 23.31 |
| NP-Of24 | *O. flavescens* | modern | -12.68 | 22.79 |
| NP-Of25 | *O. flavescens* | modern | -12.04 | 23.31 |
| NP-Of26 | *O. flavescens* | modern | -12.92 | 21.65 |
| NP-Of27 | *O. flavescens* | modern | -12.05 | 22.57 |
| NP-Of28 | *O. flavescens* | modern | -12.32 | 22.32 |
| NP-Of29 | *O. flavescens* | modern | -12.14 | 22.25 |
| NP-Of30 | *O. flavescens* | modern | -11.78 | 22.36 |
| NP-Of31 | *O. flavescens* | modern | -12.51 | 21.79 |
| NP-Of32 | *O. flavescens* | modern | -13.33 | 21.66 |
| NP-Of33 | *O. flavescens* | modern | -12.62 | 21.98 |
| NP-Of34 | *O. flavescens* | modern | -13.03 | 20.07 |
| NP-Of35 | *O. flavescens* | modern | -12.99 | 22.39 |
| NP-Of36 | *O. flavescens* | modern | -13.13 | 21.00 |
| NP-Sm1 | *S. magellanicus* | modern | -15.00 | 19.40 |
| NP-Sm2 | *S. magellanicus* | modern | -15.00 | 17.80 |
| NP-Sm3 | *S. magellanicus* | modern | -15.30 | 17.90 |
| NP-Sm4 | *S. magellanicus* | modern | -17.00 | 17.90 |
| NP-Sm5 | *S. magellanicus* | modern | -16.00 | 17.20 |
| NP-Sm6 | *S. magellanicus* | modern | -14.80 | 20.20 |
| NP-Sm7 | *S. magellanicus* | modern | -14.80 | 20.30 |
| NP-Sm8 | *S. magellanicus* | modern | -15.10 | 20.40 |
| NP-Sm9 | *S. magellanicus* | modern | -14.60 | 19.30 |
| NP-Sm10 | *S. magellanicus* | modern | -13.40 | 18.40 |
| NP-Sm11 | *S. magellanicus* | modern | -15.10 | 19.70 |
| NP-Sm12 | *S. magellanicus* | modern | -15.30 | 20.20 |
| NP-Sm13 | *S. magellanicus* | modern | -13.90 | 14.50 |
| NP-Sm14 | *S. magellanicus* | modern | -15.50 | 20.10 |
| NP-Sm15 | *S. magellanicus* | modern | -14.30 | 19.40 |
| NP-Sm16 | *S. magellanicus* | modern | -15.00 | 21.00 |
| NP-Sm17 | *S. magellanicus* | modern | -17.20 | 19.60 |
| NP-Sm18 | *S. magellanicus* | modern | -14.10 | 20.60 |
| NP-Sm19 | *S. magellanicus* | modern | -15.30 | 20.20 |
| NP-Sm20 | *S. magellanicus* | modern | -13.90 | 14.50 |
| A7-cC1 | Herbivore | pre-contact | -14.74 | 13.79 |
| A7-cC4 | Herbivore | pre-contact | -14.31 | 13.71 |
| A7-cC5 | Herbivore | pre-contact | -14.74 | 13.67 |
| A8-cC2 | Herbivore | pre-contact | -18.59 | 13.29 |
| A8-cC4 | Herbivore | pre-contact | -13.79 | 13.30 |
| A5-cC1 | Herbivore | pre-contact | -14.96 | 11.93 |
| A5-cC2 | Herbivore | pre-contact | -13.73 | 13.11 |
| A5-cC3 | Herbivore | pre-contact | -15.97 | 15.83 |
| A5-cC4 | Herbivore | pre-contact | -14.00 | 13.79 |
| A5-cC5 | Herbivore | pre-contact | -12.95 | 13.88 |
| A11-cC3 | Herbivore | pre-contact | -17.91 | 14.06 |
| A11-cC4 | Herbivore | pre-contact | -17.91 | 14.21 |
| A7-cL1 | Herbivore | pre-contact | -15.74 | 16.19 |
| A7-cL2 | Herbivore | pre-contact | -14.01 | 15.03 |
| A7-cL3 | Herbivore | pre-contact | -14.28 | 16.49 |
| A7-cL4 | Herbivore | pre-contact | -14.35 | 14.94 |
| A7-cL5 | Herbivore | pre-contact | -13.84 | 14.83 |
| A10-cL1 | Herbivore | pre-contact | -12.28 | 14.40 |
| A10-cL2 | Herbivore | pre-contact | -17.16 | 13.28 |
| A10-cL3 | Herbivore | pre-contact | -12.10 | 12.10 |
| A10-cL4 | Herbivore | pre-contact | -16.33 | 14.39 |
| A10-cL5 | Herbivore | pre-contact | -17.39 | 13.58 |
| A4-cL1 | Herbivore | pre-contact | -11.39 | 15.31 |
| A4-cL3 | Herbivore | pre-contact | -15.85 | 16.27 |
| A4-cL4 | Herbivore | pre-contact | -17.72 | 15.81 |
| A4-cL5 | Herbivore | pre-contact | -15.80 | 15.02 |
| A11-cL1 | Herbivore | pre-contact | -17.81 | 13.24 |
| A11-cL2 | Herbivore | pre-contact | -17.30 | 15.05 |
| N° 90 | *A. australis* | pre-contact | -13.68 | 16.83 |
| N° 91 | *A. australis* | pre-contact | -14.45 | 17.83 |
| F1 18 | *A. australis* | pre-contact | -12.96 | 22.09 |
| F1 19 | *A. australis* | pre-contact | -12.69 | 17.16 |
| FM1 11 | *A. australis* | pre-contact | -12.52 | 18.36 |
| 36 | *O. flavescens* | pre-contact | -12.29 | 21.38 |
| 82 | *O. flavescens* | pre-contact | -11.78 | 23.82 |
| i 1 (61) | *O. flavescens* | pre-contact | -13.11 | 21.27 |
| i 3 (43) | *O. flavescens* | pre-contact | -13.04 | 22.01 |
| i 4 (44) | *O. flavescens* | pre-contact | -13.37 | 22.28 |
| i 5 (54) | *O. flavescens* | pre-contact | -10.99 | 22.24 |
| i 15 (C1) | *O. flavescens* | pre-contact | -14.37 | 22.25 |
| i 18 (89) | *O. flavescens* | pre-contact | -13.60 | 24.97 |
| i 23 | *O. flavescens* | pre-contact | -12.83 | 21.29 |
| i 24 | *O. flavescens* | pre-contact | -14.56 | 23.20 |
| M1 1 | *O. flavescens* | pre-contact | -12.04 | 22.32 |
| M1 3 | *O. flavescens* | pre-contact | -12.96 | 22.41 |
| FSM-SRH-Mont II OF (costilla) | *O. flavescens* | pre-contact | -11.41 | 22.40 |
| OBS 13 | *O. flavescens* | pre-contact | -11.25 | 22.41 |
| FSM-SRH-Mont I OF (cost px med) | *O. flavescens* | pre-contact | -11.91 | 22.98 |
| OBS 4 | *O. flavescens* | pre-contact | -13.89 | 18.06 |
| OBS 137 | *O. flavescens* | pre-contact | -13.45 | 21.10 |
| F1 17 | *O. flavescens* | pre-contact | -13.67 | 19.89 |
| FM1 13 | *O. flavescens* | pre-contact | -13.10 | 19.74 |
| FM1 14 | *O. flavescens* | pre-contact | -13.37 | 20.98 |
| FM1 15 | *O. flavescens* | pre-contact | -12.06 | 20.31 |
| FM1 16 | *O. flavescens* | pre-contact | -12.31 | 18.70 |
| FM1 17 | *O. flavescens* | pre-contact | -12.70 | 18.92 |
| FM1 19 | *O. flavescens* | pre-contact | -12.90 | 22.47 |
| FM1 20 | *O. flavescens* | pre-contact | -12.27 | 21.90 |
| FM1 21 | *O. flavescens* | pre-contact | -12.78 | 20.32 |
| OBS 26 | *S. magellanicus* | pre-contact | -13.13 | 17.43 |
| 7 | *S. magellanicus* | pre-contact | -14.93 | 16.44 |
| 72 | *S. magellanicus* | pre-contact | -10.68 | 15.14 |
| 80 | *S. magellanicus* | pre-contact | -13.27 | 20.79 |
| 82 | *S. magellanicus* | pre-contact | -13.10 | 20.46 |
| FSM-S2N3-Obs26 (tibia derecha) | *S. magellanicus* | pre-contact | -13.18 | 18.19 |
| FSM-S2N2-Obs21 (cráneo) | *S. magellanicus* | pre-contact | -13.39 | 18.85 |
