# Supplementary material for: Longer and Less Overlapping Food Webs in Anthropogenically Disturbed Marine Ecosystems: Confirmations from the Past
Source: PLoS One. 2014 Jul 30;9(7):e103132. doi: 10.1371/journal.pone.0103132 (PMC4116168; doi:10.1371/journal.pone.0103132)
Supplement: Table S3 — Modern and archaeological nitrogen and carbon stable-isotope ratios in southern Patagonia. Table shows the list of samples, grouped according to their historical period (modern, LAP and EAP), and δ13C and δ15N values. (DOCX) [file pone.0103132.s003.docx]

| **Sample** | **Species/Group** | **Period** | **δ^13^C** | **δ^15^N** |
| --- | --- | --- | --- | --- |
| SP-H1 | Herbivore | Modern | -17.62 | 12.20 |
| SP-H2 | Herbivore | Modern | -16.54 | 11.76 |
| SP-H3 | Herbivore | Modern | -16.40 | 11.52 |
| SP-H4 | Herbivore | Modern | -17.10 | 11.85 |
| SP-H5 | Herbivore | Modern | -16.01 | 12.01 |
| SP-H6 | Herbivore | Modern | -10.40 | 10.90 |
| SP-H7 | Herbivore | Modern | -12.00 | 10.37 |
| SP-H8 | Herbivore | Modern | -13.50 | 10.90 |
| SP-H9 | Herbivore | Modern | -12.50 | 10.70 |
| SP-H10 | Herbivore | Modern | -14.80 | 11.00 |
| SP-Aa1 | *A. australis* | Modern | -13.70 | 21.27 |
| SP-Aa2 | *A. australis* | Modern | -13.69 | 18.48 |
| SP-Aa3 | *A. australis* | Modern | -14.08 | 16.69 |
| SP-Aa4 | *A. australis* | Modern | -12.20 | 18.15 |
| SP-Aa5 | *A. australis* | Modern | -12.54 | 21.44 |
| SP-Aa6 | *A. australis* | Modern | -13.63 | 19.41 |
| SP-Aa7 | *A. australis* | Modern | -13.32 | 19.96 |
| SP-Of1 | *O.flavescens* | Modern | -12.10 | 22.20 |
| SP-Of2 | *O.flavescens* | Modern | -11.90 | 21.80 |
| SP-Of3 | *O.flavescens* | Modern | -13.10 | 21.60 |
| SP-Of4 | *O.flavescens* | Modern | -11.70 | 22.20 |
| SP-Of5 | *O.flavescens* | Modern | -11.65 | 20.11 |
| SP-Of6 | *O.flavescens* | Modern | -11.66 | 19.68 |
| SP-Of7 | *O.flavescens* | Modern | -11.62 | 19.88 |
| SP-Of8 | *O.flavescens* | Modern | -12.69 | 20.69 |
| SP-Of9 | *O.flavescens* | Modern | -13.83 | 20.65 |
| SP-Of10 | *O.flavescens* | Modern | -12.35 | 20.33 |
| SP-Of11 | *O.flavescens* | Modern | -11.40 | 19.10 |
| SP-Of12 | *O.flavescens* | Modern | -11.00 | 22.40 |
| SP-Of13 | *O.flavescens* | Modern | -13.73 | 21.28 |
| SP-Of14 | *O.flavescens* | Modern | -13.20 | 18.50 |
| SP-Of15 | *O.flavescens* | Modern | -12.50 | 18.20 |
| SP-Of16 | *O.flavescens* | Modern | -12.90 | 19.70 |
| SP-Of17 | *O.flavescens* | Modern | -11.60 | 22.40 |
| SP-Of18 | *O.flavescens* | Modern | -14.90 | 20.80 |
| SP-Of19 | *O.flavescens* | Modern | -12.99 | 18.89 |
| SP-Of20 | *O.flavescens* | Modern | -12.54 | 20.68 |
| SP-Of21 | *O.flavescens* | Modern | -12.70 | 20.20 |
| SP-Of22 | *O.flavescens* | Modern | -14.65 | 20.30 |
| SP-Of23 | *O.flavescens* | Modern | -12.50 | 21.30 |
| SP-Of24 | *O.flavescens* | Modern | -13.80 | 21.00 |
| SP-Of25 | *O.flavescens* | Modern | -13.40 | 20.90 |
| SP-Of26 | *O.flavescens* | Modern | -12.40 | 21.20 |
| SP-Of27 | *O.flavescens* | Modern | -12.00 | 20.90 |
| SP-Of28 | *O.flavescens* | Modern | -13.60 | 20.50 |
| SP-Of29 | *O.flavescens* | Modern | -13.00 | 21.20 |
| SP-Of30 | *O.flavescens* | Modern | -13.20 | 20.60 |
| SP-Of31 | *O.flavescens* | Modern | -13.25 | 20.19 |
| SP-Of32 | *O.flavescens* | Modern | -13.61 | 20.14 |
| SP-Of33 | *O.flavescens* | Modern | -12.52 | 20.50 |
| SP-Of34 | *O.flavescens* | Modern | -13.50 | 20.70 |
| SP-Of35 | *O.flavescens* | Modern | -13.10 | 20.60 |
| SP-Of36 | *O.flavescens* | Modern | -13.10 | 20.70 |
| SP-Of37 | *O.flavescens* | Modern | -13.42 | 21.07 |
| SP-Of38 | *O.flavescens* | Modern | -16.83 | 23.23 |
| SP-Of39 | *O.flavescens* | Modern | -13.65 | 20.66 |
| SP-Of40 | *O.flavescens* | Modern | -13.65 | 20.93 |
| SP-Of41 | *O.flavescens* | Modern | -12.30 | 23.40 |
| SP-Sm1 | *S. magellanicus* | Modern | -15.40 | 19.50 |
| SP-Sm2 | *S. magellanicus* | Modern | -16.50 | 18.10 |
| SP-Sm3 | *S. magellanicus* | Modern | -14.50 | 19.70 |
| SP-Sm4 | *S. magellanicus* | Modern | -17.10 | 19.00 |
| SP-Sm5 | *S. magellanicus* | Modern | -14.80 | 19.00 |
| SP-Sm6 | *S. magellanicus* | Modern | -15.10 | 20.10 |
| SP-Sm7 | *S. magellanicus* | Modern | -16.30 | 18.80 |
| SP-Sm8 | *S. magellanicus* | Modern | -14.80 | 18.80 |
| SP-Sm9 | *S. magellanicus* | Modern | -13.90 | 19.10 |
| SP-Sm10 | *S. magellanicus* | Modern | -15.50 | 19.50 |
| SP-Sm11 | *S. magellanicus* | Modern | -17.10 | 18.40 |
| SP-Sm12 | *S. magellanicus* | Modern | -16.90 | 19.50 |
| SP-Sm13 | *S. magellanicus* | Modern | -16.50 | 19.10 |
| SP-Sm14 | *S. magellanicus* | Modern | -17.00 | 19.20 |
| SP-Sm15 | *S. magellanicus* | Modern | -14.90 | 19.70 |
| SP-Sm16 | *S. magellanicus* | Modern | -16.20 | 19.30 |
| SP-Sm17 | *S. magellanicus* | Modern | -15.50 | 19.60 |
| SP-Sm18 | *S. magellanicus* | Modern | -16.00 | 19.40 |
| SP-Sm19 | *S. magellanicus* | Modern | -15.90 | 19.10 |
| SP-Sm20 | *S. magellanicus* | Modern | -15.90 | 18.80 |
| SP-Sm21 | *S. magellanicus* | Modern | -15.90 | 19.20 |
| SP-Sm22 | *S. magellanicus* | Modern | -15.20 | 20.30 |
| SP-Sm23 | *S. magellanicus* | Modern | -15.50 | 19.90 |
| SP-Sm24 | *S. magellanicus* | Modern | -14.80 | 20.00 |
| SP-Sm25 | *S. magellanicus* | Modern | -14.90 | 18.70 |
| SP-Sm26 | *S. magellanicus* | Modern | -14.60 | 19.10 |
| SP-Sm27 | *S. magellanicus* | Modern | -14.90 | 19.00 |
| SP-Sm28 | *S. magellanicus* | Modern | -14.60 | 19.10 |
| SP-Sm29 | *S. magellanicus* | Modern | -14.00 | 19.70 |
| SP-Sm30 | *S. magellanicus* | Modern | -14.30 | 19.50 |
| SP-Sm31 | *S. magellanicus* | Modern | -14.70 | 19.60 |
| SP-Sm32 | *S. magellanicus* | Modern | -14.70 | 18.50 |
| SP-Sm33 | *S. magellanicus* | Modern | -14.80 | 19.50 |
| SP-Sm34 | *S. magellanicus* | Modern | -14.40 | 19.10 |
| SP-Sm35 | *S. magellanicus* | Modern | -14.50 | 19.50 |
| SP-Sm36 | *S. magellanicus* | Modern | -15.00 | 19.70 |
| SP-Sm37 | *S. magellanicus* | Modern | -14.90 | 19.40 |
| SP-Sm38 | *S. magellanicus* | Modern | -17.30 | 20.00 |
| SP-Sm39 | *S. magellanicus* | Modern | -16.50 | 19.30 |
| SP-Sm40 | *S. magellanicus* | Modern | -17.10 | 18.10 |
| A17-cM1 | Herbivore | LAP | -20.07 | 13.01 |
| A17-cM2 | Herbivore | LAP | -19.59 | 13.32 |
| A17-cM3 | Herbivore | LAP | -20.27 | 14.30 |
| A17-cM4 | Herbivore | LAP | -21.35 | 14.01 |
| A17-cM5 | Herbivore | LAP | -21.09 | 12.15 |
| A15-cM1 | Herbivore | LAP | -14.32 | 12.86 |
| A15-cM2 | Herbivore | LAP | -14.99 | 13.43 |
| A15-cM3 | Herbivore | LAP | -14.53 | 13.72 |
| A17-cL1 | Herbivore | LAP | -16.74 | 13.11 |
| A17-cL2 | Herbivore | LAP | -17.35 | 12.00 |
| A17-cL3 | Herbivore | LAP | -20.99 | 13.02 |
| A17-cL4 | Herbivore | LAP | -22.15 | 11.73 |
| A17-cL5 | Herbivore | LAP | -19.52 | 12.59 |
| A15-cL2 | Herbivore | LAP | -11.44 | 11.17 |
| A15-cL4 | Herbivore | LAP | -14.52 | 12.66 |
| 44331 | *A. australis* | LAP | -12.00 | 16.80 |
| 155288 | *A. australis* | LAP | -11.70 | 17.46 |
| 150329 | *A. australis* | LAP | -12.10 | 18.06 |
| 152253 | *A. australis* | LAP | -11.60 | 16.71 |
| 152439 | *A. australis* | LAP | -11.70 | 17.16 |
| 151607 | *A. australis* | LAP | -12.00 | 16.13 |
| 154656 | *A. australis* | LAP | -11.70 | 17.49 |
| 151575 | *A. australis* | LAP | -11.80 | 17.01 |
| 151912 | *A. australis* | LAP | -12.60 | 16.77 |
| 154284 | *A. australis* | LAP | -12.40 | 17.22 |
| 153887 | *A. australis* | LAP | -11.50 | 17.88 |
| 155456 | *A. australis* | LAP | -12.30 | 15.46 |
| 155447 | *A. australis* | LAP | -11.60 | 17.15 |
| OF 2a | *O.flavescens* | LAP | -13.81 | 17.40 |
| OF 2b | *O.flavescens* | LAP | -15.65 | 16.81 |
| OF 3 | *O.flavescens* | LAP | -15.12 | 16.52 |
| OF 9 | *O.flavescens* | LAP | -14.24 | 16.31 |
| OF 12 | *O.flavescens* | LAP | -12.23 | 17.81 |
| OF 14 | *O.flavescens* | LAP | -13.17 | 17.81 |
| OF 15 | *O.flavescens* | LAP | -14.12 | 19.23 |
| OF 5 | *O.flavescens* | LAP | -15.65 | 16.92 |
| OF 7 | *O.flavescens* | LAP | -12.62 | 17.32 |
| OF 10 | *O.flavescens* | LAP | -12.62 | 17.32 |
| 43418 | *O.flavescens* | LAP | -11.70 | 18.50 |
| 154286 | *O.flavescens* | LAP | -11.30 | 17.67 |
| 10030 | *S. magellanicus* | LAP | -12.38 | 16.89 |
| 10100 | *S. magellanicus* | LAP | -12.96 | 15.42 |
| pingüino 4b | *S. magellanicus* | LAP | -13.32 | 17.35 |
| pingüino 6 | *S. magellanicus* | LAP | -14.32 | 18.22 |
| pingüino 4a | *S. magellanicus* | LAP | -15.59 | 17.14 |
| 9255 | *S. magellanicus* | LAP | -12.79 | 17.61 |
| 19098 | *S. magellanicus* | LAP | -14.70 | 16.00 |
| 19264 | *S. magellanicus* | LAP | -14.07 | 17.30 |
| 10122 | *S. magellanicus* | LAP | -14.26 | 17.41 |
| 12433 | *S. magellanicus* | LAP | -14.82 | 18.20 |
| 12268 | *S. magellanicus* | LAP | -15.84 | 17.55 |
| 10116 | *S. magellanicus* | LAP | -15.92 | 17.72 |
| 10115 | *S. magellanicus* | LAP | -16.63 | 17.46 |
| A19-cM1 | Herbivore | EAP | -22.34 | 15.06 |
| A19-cM2 | Herbivore | EAP | -17.07 | 14.35 |
| A19-cM3 | Herbivore | EAP | -17.25 | 16.95 |
| A19-cM4 | Herbivore | EAP | -17.09 | 16.28 |
| A19-cM5 | Herbivore | EAP | -17.40 | 14.30 |
| A19-cL1 | Herbivore | EAP | -18.40 | 12.81 |
| A19-cL2 | Herbivore | EAP | -19.79 | 13.61 |
| A19-cL3 | Herbivore | EAP | -20.43 | 13.32 |
| A19-cL4 | Herbivore | EAP | -15.05 | 14.19 |
| A19-cL5 | Herbivore | EAP | -18.33 | 13.03 |
| CV6 4/-45-50cm | *A. australis* | EAP | -12.96 | 20.47 |
| CdN2-0072 | *A. australis* | EAP | -12.82 | 19.35 |
| 37295 | *A. australis* | EAP | -11.70 | 18.68 |
| 37340 | *A. australis* | EAP | -12.80 | 18.29 |
| 37456 | *A. australis* | EAP | -12.00 | 16.17 |
| 67319 | *A. australis* | EAP | -10.90 | 17.45 |
| 66397 | *A. australis* | EAP | -11.40 | 16.75 |
| 186854 | *A. australis* | EAP | -12.10 | 16.99 |
| 65989 | *A. australis* | EAP | -12.20 | 17.13 |
| 202401 | *A. australis* | EAP | -10.90 | 17.41 |
| 202083 | *A. australis* | EAP | -11.80 | 16.36 |
| 193261 | *A. australis* | EAP | -11.40 | 18.06 |
| 194047 | *A. australis* | EAP | -12.10 | 17.22 |
| 43247 | *A. australis* | EAP | -11.80 | 17.38 |
| 174498 | *A. australis* | EAP | -11.90 | 16.36 |
| 217933 | *A. australis* | EAP | -12.40 | 17.89 |
| 215241 | *A. australis* | EAP | -12.70 | 17.38 |
| 223614 | *A. australis* | EAP | -11.60 | 16.80 |
| 215940/215933 | *A. australis* | EAP | -11.60 | 17.33 |
| 53580 | *A. australis* | EAP | -11.20 | 17.56 |
| 68445 | *A. australis* | EAP | -10.90 | 18.12 |
| 64460 | *A. australis* | EAP | -11.70 | 17.22 |
| 213370 | *A. australis* | EAP | -11.50 | 17.67 |
| 215074 | *A. australis* | EAP | -12.20 | 17.29 |
| 58630 | *A. australis* | EAP | -12.40 | 18.11 |
| 190846 | *A. australis* | EAP | -12.00 | 17.68 |
| 63330 | *A. australis* | EAP | -11.30 | 18.14 |
| 213732 | *A. australis* | EAP | -11.50 | 17.88 |
| 52463 | *A. australis* | EAP | -12.00 | 17.65 |
| 226119 | *A. australis* | EAP | -10.80 | 17.15 |
| 69639 | *A. australis* | EAP | -11.50 | 17.29 |
| 189603 | *A. australis* | EAP | -12.50 | 17.54 |
| 216713 | *A. australis* | EAP | -11.90 | 17.38 |
| 212616/212653 | *A. australis* | EAP | -12.40 | 17.24 |
| 224151 | *A. australis* | EAP | -12.40 | 17.99 |
| 30459 | *O.flavescens* | EAP | -11.27 | 19.63 |
| 33459 | *O.flavescens* | EAP | -11.45 | 18.72 |
| 33551 | *O.flavescens* | EAP | -11.80 | 18.78 |
| 33571 | *O.flavescens* | EAP | -12.46 | 19.04 |
| 33717 | *O.flavescens* | EAP | -12.67 | 18.37 |
| 34177 | *O.flavescens* | EAP | -12.33 | 18.58 |
| 34544 | *O.flavescens* | EAP | -12.00 | 18.39 |
| 34751 | *O.flavescens* | EAP | -11.61 | 18.86 |
| 33458 | *O.flavescens* | EAP | -12.37 | 18.51 |
| CV6 4/-60-65cm | *O.flavescens* | EAP | -13.92 | 20.75 |
| CV20 OF1 | *O.flavescens* | EAP | -14.80 | 15.41 |
| CV20 OF3 | *O.flavescens* | EAP | -14.50 | 17.95 |
| CV20 OF4 | *O.flavescens* | EAP | -12.25 | 19.11 |
| CV20 OF6 | *O.flavescens* | EAP | -12.29 | 19.55 |
| CV20 OF7 | *O.flavescens* | EAP | -14.16 | 18.84 |
| 1925 | *S. magellanicus* | EAP | -14.86 | 17.46 |
| 26006 | *S. magellanicus* | EAP | -13.35 | 17.70 |
| 27597 | *S. magellanicus* | EAP | -15.32 | 16.92 |
| 3761 | *S. magellanicus* | EAP | -13.90 | 18.31 |
| 4179 | *S. magellanicus* | EAP | -14.54 | 17.79 |
| 3522 | *S. magellanicus* | EAP | -15.29 | 17.39 |
| 3641 | *S. magellanicus* | EAP | -14.98 | 18.65 |
